# Diagnosis of common health conditions among autistic adults in the UK: evidence from a matched cohort study

**DOI:** 10.1016/j.lanepe.2024.100907

**Published:** 2024-05-03

**Authors:** Elizabeth O'Nions, Jude Brown, Joshua E.J. Buckman, Rebecca Charlton, Claudia Cooper, Céline El Baou, Francesca Happé, Sarah Hoare, Dan Lewer, Jill Manthorpe, Douglas G.J. McKechnie, Marcus Richards, Rob Saunders, Will Mandy, Joshua Stott

**Affiliations:** a UCL Research Department of Clinical, Educational and Health Psychology, Division of Psychology and Language Sciences, University College London, 1 – 19 Torrington Place, London, WC1E 7HB, UK; b Bradford Institute for Health Research, Bradford Teaching Hospitals NHS Foundation Trust, Duckworth Lane, Bradford, BD9 6RJ, UK; c National Autistic Society, 391-393 City Rd, London, EC1V 1NG, UK; d iCope – Camden & Islington NHS Foundation Trust, St Pancras Hospital, London, NW1 0PE, UK; e Department of Psychology, Goldsmiths, University of London, New Cross, London, SE14 6NW, UK; f Queen Mary, University of London, Centre for Psychiatry and Mental Health, Wolfson Institute of Population Health, London, E1 2AD, UK; g Social, Genetic and Developmental Psychiatry Centre, Institute of Psychiatry, Psychology and Neuroscience, King's College London, Memory Lane, London, SE5 8AF, UK; h Institute of Epidemiology and Healthcare, University College London, 1-19 Torrington Place, London, WC1E 7HB, UK; i NIHR Policy Research Unit in Health & Social Care Workforce, King's College London, Strand, London, WC2R 2LS, UK; j UCL Research Department of Primary Care and Population Health, UCL Medical School (Royal Free Campus), Rowland Hill Street, London, NW3 2PF, UK; k MRC Unit for Lifelong Health and Ageing at UCL, 1-19 Torrington Place, London, WC1E 7HB, UK

**Keywords:** Autism, Intellectual disability, Primary care, Mental health, Substance use, Physical health

## Abstract

**Background:**

Autistic people are disproportionately likely to experience premature mortality and most mental and physical health conditions. We measured the incidence of diagnosed conditions accounting for the most disability-adjusted life years in the UK population according to the Global Burden of Disease study (anxiety, depression, self-harm, harmful alcohol use, substance use, migraine, neck or back pain, and gynaecological conditions).

**Methods:**

Participants were aged 18 years or above and had an autism diagnosis recorded in the IQVIA Medical Research Database between 01/01/2000 and 16/01/2019. We included 15,675 autistic adults without intellectual disability, 6437 autistic adults with intellectual disability, and a comparison group matched (1:10) by age, sex, and primary care practice. We estimated crude incidences and incidence rate ratios (IRRs) adjusted for age and sex.

**Findings:**

Autistic adults without intellectual disability experienced a higher incidence (IRR, 95% CI) of self-harm (2.07, 1.79–2.40), anxiety (1.91, 1.76–2.06), depressive disorders (1.79, 1.67–1.92), and substance use (1.24, 1.02–1.51) relative to comparison participants. Incidences of harmful alcohol use (1.01, 0.85–1.18), migraine (0.99, 0.84–1.17), and gynaecological conditions (1.19, 0.95–1.49) did not differ. Neck or back pain incidence was lower (0.88, 0.82–0.95). Autistic adults with intellectual disability experienced a higher incidence of self-harm (2.08, 1.69–2.56). Incidences of anxiety (1.14, 1.00–1.30), gynaecological conditions (1.22, 0.93–1.62), and substance use (1.08, 0.80–1.47) did not differ, and lower incidences were found for depressive disorders (0.73, 0.64–0.83), harmful alcohol use (0.65, 0.50–0.84), migraine (0.55, 0.42–0.74), and neck or back pain (0.49, 0.44–0.55).

**Interpretation:**

Although our findings cannot be directly compared to previous prevalence studies, they contrast with the higher frequency of mental and physical health conditions in autistic adults reported in studies that directly assessed and/or surveyed autistic people about co-occurring conditions. The present findings may suggest under-diagnosis of common conditions in autistic people, particularly those with intellectual disability. Improved detection should be a clinical and policy priority to reduce health inequalities.

**Funding:**

10.13039/501100000377Dunhill Medical Trust, 10.13039/501100000269Economic and Social Research Council, National Institute of Health and Care Research.

**Research in context**

**Evidence before this study**

We searched PubMed from database inception to January 23rd, 2024 using the search terms: (1) ‘autis∗’, and (2) ‘mental health’, ‘physical health’, plus each of the studied conditions, without language restrictions. This identified articles describing the frequency of mental and physical health conditions in autistic and comparison people. Some studies identified rates based on routinely-collected data, and others actively assessed or surveyed participants or their supporters about their health. The vast majority of studies focused on mental rather than physical health conditions. None of the identified studies compared the incidence of diagnosed mental or physical health conditions in a population-based sample of diagnosed autistic people with and without intellectual disability aged 18 years and above to rates in a matched comparison group.

**Added value of this study**

This study is the first to compare the rates of new diagnoses of common conditions responsible for the most disability-adjusted life years in United Kingdom (UK) between diagnosed autistic adults aged 18 years and above with a population-based comparison group. The rate of incident self-harm (including self-injury) was more than twice as high in autistic people with and without intellectual disability than in the general population, suggesting disproportionately unmet mental, physical, or sensory-related support needs. For autistic people without intellectual disability, the rate difference for new anxiety and depression diagnoses relative to the comparison group was not as high as was expected given evidence that autistic people experience a much higher frequency of these conditions in studies that assess them actively. Comparable rates of diagnosed migraine and gynaecological conditions, and lower rates of diagnosed neck/back pain could also indicate under-diagnosis, given the evidence from survey-based studies that these conditions are more common in autistic people. For autistic people with intellectual disability, comparable or lower-than-average rates of new diagnoses of mental health conditions were found. Rates of new diagnoses of migraine, neck or back pain, and gynaecological conditions (when codes indexing premenstrual dysphoria were excluded) were substantially lower. This is despite the much higher rate of diagnosed epilepsy and severe mobility problems, which were expected to increase the rate of migraine and neck and back pain respectively.

**Implications of all the available evidence**

Our results are not directly comparable with those from previous studies, many of which report the rates of particular health conditions based on a survey or assessment. However, our findings may suggest that common conditions are under-diagnosed among autistic people. Studies in which symptoms are actively measured and compared to rates of diagnoses are needed to confirm this. However, viewed in the light of other evidence indicating health inequalities, the present findings raise the possibility that potentially serious conditions may more often go undetected and untreated in autistic people, leading to greater morbidity and the known premature mortality in this group.

## Introduction

Autism is a lifelong neurodevelopmental condition present from birth, which impacts how a person relates to others and perceives the world.^m^ Diagnostic criteria include social communication and social interaction differences, plus restricted and repetitive patterns of behaviours, interests, and activities.^1^ Between 1% and 3% of the population are autistic.^2^^,^^3^ Autistic people have widely varying support needs. Studies of clinically diagnosed autistic adults suggest that around 30% have an intellectual disability,^4^ though studies using active identification of autistic people in the community suggest the proportion may be smaller.^5^ Autistic children and adults disproportionately experience disadvantage and adversity, including discrimination,^6^ social exclusion,^7^ unemployment,^8^ adverse life events,^7^^,^^9^, ^10^, ^11^, ^12^ and difficulties achieving desired social relationships^13^; which contribute to poorer health,^14^ impede healthcare access,^15^ and lead to poorer treatment outcomes.^16^^,^^17^

Autistic adults report barriers to healthcare access, such as not knowing whether a problem requires attention or where to access support, not having a supporter to help them, difficulties communicating with providers, finding healthcare settings overwhelming, and past experiences of stigma, discrimination, and inadequate care, leading to low expectations and anxiety.^15^^,^^17^, ^18^, ^19^, ^20^ Practitioner and systems-level barriers include a lack of statutory recognition of need in autistic children and adults in England,^21^ a lack of knowledge and staff training,^12^^,^^22^ diagnostic overshadowing,^17^^,^^23^ and the inflexibility of health systems to accommodate those with additional needs.^17^^,^^20^^,^^24^ These barriers can lead to delayed or misdiagnosis of health conditions, worsening health status, greater reliance on emergency care, and premature mortality.^17^^,^^20^^,^^25^^,^^26^

Surveys,^27^, ^28^, ^29^, ^30^ clinic-based,^31^ registry,^32^, ^33^, ^34^, ^35^ and medical claims database studies^36^, ^37^, ^38^ suggest that autistic young people and adults experience poorer mental health and higher rates of substance use relative to the general population. Non-communicable physical health conditions are also more prevalent, though the difference tends to be smaller than for mental health conditions.^34^^,^^36^^,^^39^ Survey data from the US indicate that parent-identified chronic pain, including headaches or other back or body pain, is nearly twice as common in autistic vs. non-autistic children (15.6% vs. 8.2%), with the highest rate in those with an additional developmental disability (19.9%).^40^ Higher rates of connective-tissue disorders or hypermobility,^41^^,^^42^ musculoskeletal pain,^41^ and gynaecological disorders,^39^ are also reported by autistic vs. comparison adults surveyed about their health; and reports indicate more physical health conditions linked to epilepsy, such as migraine.^39^^,^^43^^,^^44^ However, barriers to healthcare access may lead to under-detection of these and other conditions, resulting in similar or lower rates of diagnosed health problems relative to the general population in routine healthcare records.

The aim of the present study was to examine the rates of new diagnoses of common health conditions in autistic adults compared with the general population, using data from UK electronic primary care records. We focused on conditions that contribute most to morbidity in people aged 15–49 in the UK based on the Global Burden of Disease study because, due to trends in awareness and poor access to diagnostic services for adults, the majority of people with an autism diagnosis in the UK are aged under 50.^45^ Five mental health-related conditions (depression, anxiety, self-harm, substance use, harmful alcohol use), and three physical health conditions (migraine, neck or back pain, and gynaecological disorders), representing important drivers of young and middle-aged adults’ health-related quality of life, were included. Analyses were conducted separately for autistic adults with and without intellectual disability.

## Methods

### Study design

A matched retrospective cohort study.

### Setting

This study used UK electronic primary care health records from IQVIA Medical Research Data (IMRD). IQVIA Medical Research Data (IMRD) incorporates data from THIN, a Cegedim Database. Reference made to THIN is intended to be descriptive of the data asset licensed by IQVIA. IMRD contains anonymised electronic health records extracted directly from primary care computer systems for 794 UK primary care practices (c. 10% of all practices) and is approximately representative of the UK population.^46^

In the UK, almost all of the population are registered with a NHS primary care practice and access is free of charge.^47^ Observations and diagnoses are captured in a person's medical records using a structured system of clinical codes called “Read codes”, which are applied by primary care practitioners. Non-emergency secondary and specialist care are mostly accessed via referral by a primary care general practitioner (GP). Diagnoses made in secondary care are communicated to the patient's GP and recorded in their records by practice staff. Therefore, primary care records function as a repository for an individual's health-related information. To meet quality thresholds, all records were required to contain information about age, sex, and primary care practice; though there was some missing data on socioeconomic deprivation (see Table 1).

**Table 1:** Participant characteristics: autistic participants with and without an intellectual disability and their respective matched comparison groups.

	Autistic people without intellectual disability	Matched comparison group	Autistic people with intellectual disability	Matched comparison group
N individuals	15,675	156,750	6437	64,370
N practices	772	772	736	736
n males (%)	12,041 (76.82)	120,410 (76.82)	4840 (75.19)	48,400 (75.19)
n females (%)	3634 (23.18)	36,340 (23.18)	1597 (24.81)	15,970 (24.81)
n hearing impairment at start (%)	329 (2.10)^c^	2682 (1.71)	221 (3.43)^c^	1124 (1.75)
n severe mobility problem at start (%)	123 (0.78)^c^	382 (0.24)	276 (4.29)^c^	180 (0.28)
n severe visual impairment at start (%)	99 (0.63)^c^	431 (0.27)	128 (1.99)^c^	193 (0.30)
n epilepsy at start (%)	673 (4.29)^c^	1499 (0.96)	1383 (21.49)^c^	685 (1.06)
n genetic condition at start (%)	214 (1.37)^c^	871 (0.56)	541 (8.40)^c^	348 (0.54)
**Age at cohort entry**				
Median age at entry (IQR)	19.38 (18.00–26.49)	19.38 (18.00–26.49)	22.87 (18.73–34.25)	22.87 (18.73–34.25)
18–24 years	11,255 (71.80)	112,550 (71.80)	3720 (57.79)	37,200 (57.79)
25–34 years	2176 (13.88)	21,760 (13.88)	1187 (18.44)	11,870 (18.44)
35–44 years	1140 (7.27)	11,400 (7.27)	742 (11.53)	7420 (11.53)
45–54 years	736 (4.70)	7360 (4.70)	504 (7.83)	5040 (7.83)
55–64 years	282 (1.80)	2820 (1.80)	204 (3.17)	2040 (3.17)
65+ years	86 (0.55)	860 (0.55)	80 (1.24)	800 (1.24)
Median length of the observation period (IQR)	2.17 (0.87–4.51)	2.52 (1.02–4.85)	3.03 (1.26–6.23)	3.35 (1.37–6.35)
**Socioeconomic status**				
n Townsend score 1 (%)	2407 (15.36)	28,057 (17.90)	977 (15.18)	12,064 (18.74)
n Townsend score 2 (%)	2265 (14.45)	25,791 (16.45)	1073 (16.67)	11,258 (17.49)
n Townsend score 3 (%)	2726 (17.39)	28,429 (18.14)	1310 (20.35)	11,880 (18.46)
n Townsend score 4 (%)	2649 (16.90)	25,794 (16.46)	1012 (15.72)	10,838 (16.84)
n Townsend score 5 (%)	1944 (12.40)	17,972 (11.47)	760 (11.81)	7638 (11.87)
n Townsend score missing (%)	3684 (23.50)	30,707 (19.59)	1305 (20.27)	10,692 (16.61)
**Pre-existing conditions at start (baseline)**				
n anxiety at start (%)	3270 (20.86)^c^	9576 (6.11)	859 (13.34)^c^	4482 (6.96)
n depression at start (%)	3644 (23.25)^c^	11,983 (7.64)	827 (12.85)^c^	6309 (9.80)
n self-harm at start (%)	1531 (9.77)^c^	4050 (2.58)	424 (6.59)^c^	1979 (3.07)
n harmful alcohol at start (%)	436 (2.78)^c^	2951 (1.88)	104 (1.62)^b^	1444 (2.24)
n substance use at start (%)	445 (2.84)^c^	2101 (1.34)	77 (1.20)^b^	1014 (1.58)
n migraine at start (%)	879 (5.61)^b^	7893 (5.04)	168 (2.61)^c^	3446 (5.35)
n neck or back pain at start (%)	2261 (14.42)^c^	27,404 (17.48)	533 (8.28)^c^	13,570 (21.08)
n gynaecological conditions at start^a^ (%)	314 (8.64)^b^	2398 (6.60)	157 (9.83) n.s.	1357 (8.50)
**Year of cohort entry**				
2000–2010	3074 (19.61)	30,740 (19.61)	2126 (33.03)	21,260 (33.03)
2010–2019	12,601 (80.39)	126,010 (80.39)	4311 (66.97)	43,110 (66.97)

a Females only. For baseline variables on which the samples were unmatched: n. s.: non-significant.

b Nominally statistically significant difference at p < 0.05.

c Nominally statistically significant difference at p < 0.001. Townsend scores differed significantly for autistic people with and without intellectual disability and their respective comparison groups at p < 0.001. Age at cohort entry has a 6-month margin of error, because only year of birth is available for over-18s in IMRD. Ages are identical for matched groups because we matched by year of birth.

### Ethical approval

IMRD holds ethical approval to collect and supply data for research purposes from the NHS London—South East Research Ethics Committee (reference 18/LO/0441). Use of the IMRD for this study was obtained and approved by IQVIA World Publications Scientific Review Committee in June 2021 (reference 21SRC014).

### Study population

We included two cohorts: adults diagnosed with autism but not intellectual disability, and adults diagnosed with both autism and intellectual disability. Autism diagnoses were identified from the presence of a diagnostic label indicative of an autism spectrum condition (e.g., autism, Asperger's, pervasive developmental disorder) based on previously published studies.^48^, ^49^, ^50^ Code-lists are provided in a Supplemental file. For autistic participants, the cohort entry date (i.e. the start of their observation period) was the latest of the following dates: the date of their autism diagnosis (if there was a diagnosis of both autism and intellectual disability, the date of the later diagnosis), the date on which the primary care practice at which the participant was registered met quality criteria for electronic healthcare record-keeping (acceptable mortality recording^51^ and acceptable computer usage^52^), the participant's date of registration at the practice + 6 months, the date that the participant's primary care practice began contributing data to IMRD, and January 1, 2000. The participant's date of cohort exit (i.e. the end of their observation period) was the earliest of their date of death (if applicable), the date of their deregistration from the practice, the date that their practice no longer contributed data to IMRD, and January 16, 2019.

For each individual diagnosed autistic, we sampled ten comparison people matched to the autistic participant by age-, sex-, and primary care practice. For each individual joining the cohort who had been diagnosed autistic, we randomly sampled comparison individuals who did not have an autism or an intellectual disability diagnosis on the date of cohort entry for the corresponding autistic participant. We then assigned the comparison participant the same cohort entry date as their corresponding autistic participant. Cohort exit dates were identified in the same way as for autistic participants. eFigures 1 and 2 describe the identification of eligible autistic and comparison participants and cohort entry dates.

### Study variables

We identified the top 10 causes of disability adjusted life-years in the UK population for people aged 15–49, as measured by the Global Burden of Disease,^53^ using an interactive tool (https://vizhub.healthdata.org/gbd-compare/). These were: anxiety, depression, self-harm, harmful alcohol use, substance use, migraine, back pain, neck pain, gynaecological disorders, and other musculoskeletal conditions. These conditions were identified based on the presence of a relevant Read code in participants' medical records. We combined back and neck pain codes into one list, and excluded ‘other musculoskeletal conditions’, given that no information was available about the parameters of this category. Code-lists for the resulting 8 conditions were identified from existing sources^54^, ^55^, ^56^, ^57^ and from searches of relevant codes within the database (see Supplementary files for code-lists). For self-harm, we included codes for self-injury, overdoses, and suicide attempts or suicides. For gynaecological disorders, codes covered the major subcategories included within the Global Burden of Disease study^58^: uterine fibroids, polycystic ovarian syndrome, female infertility, endometriosis, genital prolapse, and premenstrual syndrome. As a sensitivity analysis, we re-ran the analysis of gynaecological disorders excluding premenstrual tension/dysphoria, since this indicates a state of psychological distress and hence could fall under mental health conditions.^59^ Code-lists were reviewed by clinicians and subject-matter experts within our team.

### Statistical analysis

Crude estimates of the incidence of new diagnoses of each condition were calculated by dividing the number of first diagnoses of a given condition during the observation period by the total person-time-at-risk (i.e. the duration of the observation period); for autistic people with and without intellectual disability and their respective comparison groups. Individuals who had a pre-existing record of a given condition (i.e. prevalent cases) were excluded from the analysis of the rate of new records of each condition. If participants received a new diagnosis of one of the studied conditions during the observation period, the observation period stopped for them on that date (since after this point, they could no longer receive a new diagnosis). Therefore, a maximum of one diagnosis for each participant was counted when estimating rates. The numbers of individuals with each condition at the start of the observation period (who were excluded from incidence calculations, since they had a pre-existing diagnosis) are provided in Table 1. χ^2^ tests were used to compare the number of participants in the autistic vs. comparison groups with a pre-existing record of a particular diagnosis.

Poisson regression was used to estimate confidence intervals for crude incidence, given that assumptions of the Poisson model were met due to the binary outcome studied (presence/absence of a health condition diagnosis at cohort exit). We used Poisson regression to estimate incidence rate ratios (IRRs) which compare the rate of diagnoses of each condition for autistic people with and without intellectual disability to that of their respective comparison groups. In these models, the relevant diagnosis was the dependent variable, and presence of an autism diagnosis, age (linear and quadratic terms), sex, and an offset for the log observation time were the independent variables. We included linear and quadratic terms for age because we anticipated that there might be non-linear relationships between age and the studied outcomes. Log-observation time was used because we are using a multiplicative Poisson model. For gynaecological conditions, analyses were run using data from females only. Data preparation and analysis were performed using Stata 16.

### Patient and public involvement

Autistic adults were involved in the design and conduct of this research. Four autistic adults provided consultancy via an Experts by Experience Steering Group, facilitated by the National Autistic Society (NAS). This group offered extensive feedback on planned analyses. Their feedback informed the preparation of the manuscript.

### Role of the funding source

The funders of the study had no role in study design, data collection, data analysis, data interpretation, or writing of the paper.

## Results

We identified 15,675 people with an autism diagnosis who did not have diagnosed intellectual disability at any time prior to cohort exit; 6437 people diagnosed autistic with concurrent diagnosed intellectual disability; and 9,504,311 people with no autism record at any time prior to or during the observation period (eFigure 1). Therefore, diagnosed autism prevalence was 0.23%, potentially 10 times lower than the true autism prevalence based on current diagnostic criteria.

Most autistic people with (75.2%) and without intellectual disability were male (76.8%) and aged between 18 and 24 years old at cohort entry (Table 1). Most (80.4%) autistic people without intellectual disability entered the cohort between 2010 and 2019, compared to 67.0% of autistic people with intellectual disability. The median age at cohort entry was 19.4 years (IQR: 18.0–26.5) for autistic people without intellectual disability, and 22.9 years (IQR: 18.7–34.3) for autistic people with intellectual disability. Further demographic and clinical information is provided in Table 1.

### Rates of prior diagnoses of common conditions at cohort entry

Compared to people not diagnosed autistic or with intellectual disability, a higher proportion of autistic people without intellectual disability had a prior record of anxiety (20.9% in the autistic group vs. 6.1% in the comparison group; χ^2^ (1) = 4500; p < 0.001), depression (23.3% vs. 7.6%; χ^2^ (1) = 4200; p < 0.001), self-harm (9.8% vs. 2.6%; χ^2^ (1) = 2300; p < 0.001), harmful alcohol use (2.8% vs. 1.9%; χ^2^ (1) = 59.79; p < 0.001), substance use (2.8% vs. 1.3%; χ^2^ (1) = 219.97; p < 0.001), migraine (5.6% vs. 5.0%; χ^2^ (1) = 9.66; p < 0.05), or gynaecological conditions (8.6% vs. 6.6%; χ^2^ (1) = 21.78; p < 0.001) at cohort entry. Fewer autistic people without intellectual disability had a prior record of neck or back pain (14.4% in the autistic group vs. 17.5% in the comparison group; χ^2^ (1) = 93.57; p < 0.001) compared to the control group (Table 1).

For autistic people with intellectual disability, a higher proportion had a prior record of anxiety (13.3% in the autistic group vs. 7.0% in the comparison group; χ^2^ (1) = 341.74; p < 0.001), depression (12.9% vs. 9.8%; χ^2^ (1) = 59.93; p < 0.001), self-harm (6.6% vs. 3.1%; χ^2^ (1) = 220.21; p < 0.001) at cohort entry. Rates of gynaecological conditions did not differ significantly (9.8% vs. 8.5%; χ^2^ (1) = 3.28; n.s.). Prior records of harmful alcohol use were less common (1.6% vs. 2.2%; χ^2^ (1) = 10.78; p < 0.05), as were substance use (1.2% vs. 1.6%; χ^2^ (1) = 5.54; p < 0.05), migraine (2.6% vs. 5.4%; χ^2^ (1) = 90.94; p < 0.001), and neck or back pain (8.3% vs. 21.0%; χ^2^ (1) = 601.18; p < 0.001) (Table 1).

### Incidence rates

**Fig. 1:**
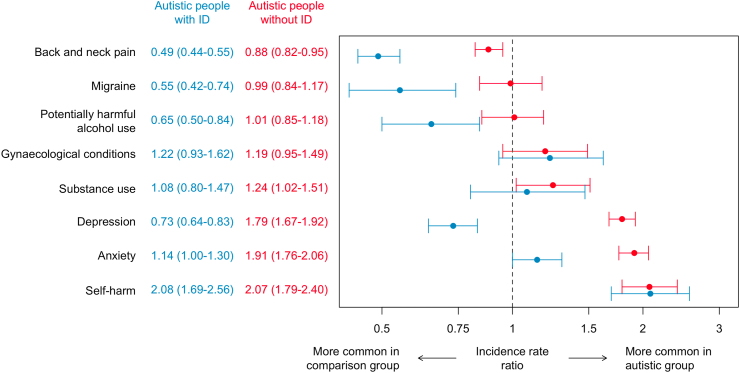
Incidence rate ratios with 95% confidence intervals comparing incidences for autistic people with and without intellectual disability (ID) to their respective matched comparison groups.

Crude incidence rates of new diagnoses of each of the conditions studied are presented in Table 2. Incidence rate ratios (IRRs) comparing autistic people with and without intellectual disability to their respective comparison groups are shown in Fig. 1. The incidence of self-harm was twice as high in autistic people without intellectual disability relative to the comparison group (IRR: 2.07, 95% CI: 1.79–2.40). Anxiety (IRR: 1.91, 95% CI: 1.76–2.06), depression (IRR: 1.79, 95% CI: 1.67–1.92), and substance use (IRR: 1.24, 95% CI: 1.02–1.51) were also higher, whilst diagnoses of neck or back pain (IRR: 0.88, 95% CI: 0.82–0.95) were lower. There was no significant difference in the incidence of harmful alcohol use (IRR: 1.01, 95% CI: 0.85–1.18), migraine (IRR: 0.99; 95% CI: 0.84–1.17), and gynaecological conditions (IRR: 1.19, 95% CI: 0.95–1.49) between the groups (Fig. 1).

**Table 2:** Crude incidence of common health conditions in autistic people with and without intellectual disability and their respective comparison groups.

New records of a given condition during the observation period	Autistic people without intellectual disability		Matched comparison group	
	N with no pre-existing record who were diagnosed during the observation period	Crude incidence per 10,000 person-years (95% CI)	N with no pre-existing record who were diagnosed during the observation period	Crude incidence per 10,000 person-years (95% CI)
**Anxiety**	**742/12,405**	**195.89 (182.05–210.50)**	**5056/147,174**	**102.08 (99.28–104.93)**
**Depression**	**948/12,031**	**263.27 (246.78–280.58)**	**6945/144,767**	**144.42 (141.05–147.86)**
**Self-harm**	**208/14,144**	**46.49 (40.39–53.26)**	**1151/152,700**	**22.00 (20.74–23.31)**
Harmful alcohol use	160/15,239	33.58 (28.58–39.20)	1773/153,799	33.84 (32.28–35.45)
**Substance use**	**110/15,230**	**23.01 (18.91–27.73)**	**963/154,649**	**18.21 (17.07–19.39)**
Migraine	157/14,796	33.91 (28.81–39.65)	1716/148,857	33.87 (32.29–35.51)
***Neck or back pain***	***846/13,414***	***211.87 (197.83–226.64)***	***9906/129,346***	***241.28 (236.55–246.08)***
Gynaecological disorders^a^	84/3320	94.27 (75.19–116.71)	806/33,942	79.89 (74.47–85.60)
	**Autistic people with intellectual disability**		**Matched comparison group**	
	N with no pre-existing record who were diagnosed during the observation period	Crude incidence per 10,000 person-years (95% CI)	N with no pre-existing record who were diagnosed during the observation period	Crude incidence per 10,000 person-years (95% CI)

Anxiety	252/5578	107.36 (94.51–121.46)	2349/59,888	92.53 (88.83–96.35)
***Depression***	***246/5610***	***106.15 (93.29–120.27)***	***3457/58,061***	***143.67 (138.92–148.54)***
**Self-harm**	**105/6013**	**40.92 (33.47–49.54)**	**530/62,391**	**19.55 (17.92–21.29)**
***Harmful alcohol use***	***61/6333***	***22.69 (17.35–29.14)***	***950/62,926***	***35.03 (32.84–37.33)***
Substance use	46/6360	17.00 (12.44–22.67)	430/63,356	15.63 (14.19–17.18)
***Migraine***	***50/6269***	***18.76 (13.93–24.74)***	***862/60,924***	***32.76 (30.61–35.02)***
***Neck or back pain***	***349/5904***	***143.29 (128.65–159.14)***	***5550/50,800***	***280.96 (273.62–288.46)***
Gynaecological disorders^a^	55/1440	93.48 (70.42–121.68)	462/14,613	75.91 (69.15–83.16)

a Females only. Rows in bold indicate conditions for which the rate was significantly higher in the autistic vs. the matched comparison group. Rows in bold and italicised indicate conditions where the rate was significantly lower in the autistic vs. the matched comparison group.

For autistic people with intellectual disability, the incidence of self-harm was twice that found in the comparison group (IRR: 2.08, 95% CI: 1.69–2.56; Fig. 1). There was no significant difference in the incidence of anxiety (IRR: 1.14, 95% CI: 1.00–1.30), substance use (IRR: 1.08, 95% CI: 0.80–1.47), and gynaecological conditions (IRR: 1.22, 95% CI: 0.93–1.62). The incidences of depression (IRR: 0.73, 95% CI: 0.64–0.83), harmful alcohol use (IRR: 0.65, 95% CI: 0.50–0.84), migraine (IRR: 0.55, 95% CI: 0.42–0.74), and neck or back pain (IRR: 0.49, 95% CI: 0.44–0.55) were lower than in the comparison group (Fig. 1).

In a sensitivity analysis, we re-ran the incidence of self-harm excluding codes pertaining to self-injurious behaviour, a term used to describe head banging, pulling at hair, picking wounds, and self-biting, which occurs in some people with intellectual disability and can be the result of sensory problems or physical discomfort. Excluding these codes, the incidence of self-harm was still elevated in both autistic people without intellectual disability (IRR: 2.04, 95% CI: 1.76–2.37) and autistic people with intellectual disability (IRR: 1.88, 95% CI: 1.51–2.34) relative to the comparison group (see eTable 1 for crude incidences). We also re-ran the incidence of self-harm excluding codes that could have pertained to accidental self-harm (e.g., drug overdoses, unless intent was specified). Excluding these codes, the incidence of self-harm was still elevated in both autistic people without intellectual disability (IRR: 2.75, 95% CI: 2.26–3.35) and autistic people with intellectual disability (IRR: 3.43, 95% CI: 2.66–4.43).

Finally, we re-ran the incidence of gynaecological conditions excluding codes for premenstrual dysphoria. Excluding this sub-group of codes, there were no differences in rates between autistic women without intellectual disability and comparison women (IRR: 0.99, 95% CI: 0.77–1.28); however, the incidence in autistic women with intellectual disability was lower than in the comparison group (IRR: 0.54, 95% CI: 0.36–0.83).

## Discussion

The aim of the present study was to compare the incidence of diagnosed common health conditions between autistic people and the general population using data from UK primary care. Autistic people with and without intellectual disability had a two-fold increased rate of self-harm (including self-injurious behaviour), suggesting poorly managed mental health and/or unmet mental, physical, and sensory-related support needs. Autistic people without intellectual disability were more likely to be newly diagnosed with a mental health condition, but autistic people with intellectual disability had a similar rate of new anxiety diagnoses and a lower rate of new depression diagnoses compared to the general population.

Despite evidence that autistic people are more likely to experience physical health conditions, rates of newly diagnosed migraine or gynaecological conditions did not differ for autistic people without intellectual disability vs. the comparison group, and rates of neck or back pain were lower. For autistic people with intellectual disability, lower incidences were found for migraine, neck or back pain, and gynaecological conditions when codes for premenstrual dysphoria were excluded.

### Comparison with other studies

Our findings cannot be directly compared to previous cohort studies that have reported on prevalence rather than incidence, but the evidence for a less-than two-fold higher rate of new diagnoses of depression and anxiety in autistic people without intellectual disability vs. the comparison group, and a similar or lower rate than the comparison group in autistic people with intellectual disability appears to contrast with evidence for a much higher frequency of mental health conditions in autistic vs. non-autistic people. Older adults self-reporting high autism characteristics in the UK were more than 7 times as likely as a population-based comparison group to score above the clinical cut-off on measures of anxiety and depression,^29^ and 5 times as likely to report self-harm with suicidal intent.^28^ Diagnosed autistic adults without intellectual disability living in England indicated a 10-fold increase in the risk of lifetime suicidal ideation when asked about this.^31^ Parent-report data from young people with intellectual disability suggests a two-fold increase in above-threshold emotional symptoms.^60^ The true prevalence of mental health conditions in people with severe-profound intellectual disability (of whom c.50% are autistic^5^) is unknown given the lack of large-scale high-quality studies,^61^ though rates of psychotropic prescribing are high,^49^^,^^62^ often motivated by the presence of behaviours that challenge.^49^ These are known to increase in the context of mood disturbances, but may have other causes.^63^

Viewed in the light of these studies where participants were explicitly surveyed about their mental health, our results suggest that depression, anxiety, and self-harm could be under-detected in autistic people with and without intellectual disability. This is concerning because diagnosing and treating depression plays a key role in preventing suicide.^64^ A recent Canadian study reported that half of autistic people with suicidal thoughts or behaviours who attended a psychiatric hospital emergency room were not identified as suicidal during initial health screenings because they initially sought support for other issues.^65^

Communication challenges are a well-documented barrier to care for autistic people.^15^ It is therefore essential that practitioners engage with autistic people to obtain information about suicidal thoughts. This may involve asking direct questions in an accessible way using unambiguous language, adapting communication to the person's preferences (e.g., offering the opportunity to communicate verbally or in writing), asking about specific symptoms rather than using diagnostic/descriptive terms, and being aware that, due to alexithymia, the person may not have self-identified as experiencing poor mental health. Identifying individuals with characteristics indicating higher risk of suicide, such as repeated self-harm, is important^64^ given the strong links between self-injury and suicide attempts in autistic people.^66^

Having a diagnosis of autism indicates that the person was assessed by a specialist psychology/psychiatry service, who may have also picked up on co-occurring mental health conditions; potentially contributing to the higher rate of diagnoses in autistic people with and without intellectual disability at baseline. At present, autistic people in England do not routinely receive post-diagnostic follow-up from specialist practitioners; therefore, incident diagnoses of common mental health conditions would usually be made by primary care practitioners. Different factors may therefore influence the rate of new vs. pre-existing mental health diagnoses; such as whether primary care practitioners elicit relevant information from autistic people.

Identifying depression in autistic people with intellectual disability may be difficult, particularly those for whom communication differences mean that they do not verbally report feelings of worthlessness or suicidal thoughts, despite experiencing changes in sleep, appetite, behaviour, and mood.^63^ The present findings suggest a need for the development and trialling of screening tools that can identify mental health problems in autistic people with intellectual disability, such as Easy Read^67^ versions of commonly used screening tools; and mental health services that are designed to be accessible to those with additional support needs.

The present results suggest that substance use could also be under-detected in autistic people. A large Swedish register-based study reported an odds-ratio of 1.6 (95% CI 1.4–1.8) for drug or alcohol misuse in autistic people, and an odds-ratio of 0.6 (95% CI 0.5–0.8) for autistic people also diagnosed with intellectual disability vs. a general population sample.^35^ However, when the data were analysed including people whose autism was identified subsequent to their drug or alcohol misuse, the odds-ratio was 2.6 (95% CI 2.4–2.9) in autistic people without intellectual disability, and 1.1 (95% CI 0.9–1.3) for those with intellectual disability vs. the comparison group.^35^ Therefore, the present findings may reflect under-detection of substance and alcohol use problems in autistic people, or under-detection of autism in people who use substances. Diagnostic overshadowing of a person's autism by their substance use problems, and a perception that autistic people are not susceptible to substance use problems may contribute to this.^35^ Substance use has been linked to risk of exploitation and homelessness, both of which are known to be more common in autistic than non-autistic people.^9^^,^^68^^,^^69^ Greater awareness of substance use among autistic people is needed to reduce the possibility of under-identification.

### Physical health

Migraine and gynaecological conditions were newly diagnosed at a similar rate for autistic people without intellectual disability and the comparison group, but the rate of new diagnoses of neck or back pain was lower. Migraine, neck or back pain, and gynaecological conditions (when premenstrual dysphoria was excluded) were all newly diagnosed at a lower rate in autistic people with intellectual disability relative to the comparison group. This was surprising given the evidence that diagnosed autistic people, particularly those with intellectual disability, have a higher frequency of physical health problems, including epilepsy, joint hypermobility, Duchenne muscular dystrophy, cardiac, and gastrointestinal disorders than the general population. These conditions are themselves associated with musculoskeletal pain^70^, ^71^, ^72^ and migraine.^73^^,^^74^ Relative to the matched comparison group, a considerably higher proportion of autistic participants with intellectual disability had epilepsy/seizure (21.5% vs. 1.1%), genetic conditions (8.4% vs. 0.5%) or severe mobility problems/cerebral palsy (4.3% vs. 0.3%) (see Table 1); suggesting that the true prevalence of migraine and neck/back pain is very likely elevated relative to the comparison group in these participants. This is also evidenced by survey and healthcare records data findings from other settings.^40^^,^^43^^,^^44^

In terms of gynaecological conditions, previous studies have reported associations between autistic traits and hyperandrogenism, reproductive system diagnoses, and excessive menstrual symptoms.^75^ Patients with PCOS-induced infertility (related to hyperandrogenism) have higher levels of autistic traits, especially social differences, compared to those with fallopian tube factor-induced infertility.^76^ In a clinically ascertained sample, significantly more autistic women reported hirsutism (indicative of hyperandrogenism), menstrual irregularities, dysmenorrhea, PCOS (polycystic ovarian syndrome), and a family history of ovarian, uterine, and prostate cancers compared to population-based comparison women.^77^ A study of 415 autistic and 415 comparison women reported higher frequencies of amenorrhea, dysmenorrhea, also suggestive of differences in steroid hormones.^78^ A study using UK primary care data reported a higher rate of PCOS in autistic women (OR: 2.01, 95% CI: 1.22–3.30).^79^

In the present study, where codes were identified from their presence in the GBD survey, the gynaecological conditions list included infertility and prolapse, which may be under-identified or less prevalent among people who do not try to become, or do not become, pregnant. Although there is a paucity of data on rates of reproduction in diagnosed autistic people, there is evidence that rates may be lower in people with intellectual disability: a 2003 survey of 2898 people with intellectual disability reported that 7% had a child, which is less than the rate in the non-intellectual disability population.^80^ Therefore, it is not clear whether our findings indicate under-diagnosis of gynaecological conditions in autistic women with intellectual disability, or a true difference in prevalence.

### Implications

The possibility that at least some common physical health conditions studied here may be under-diagnosed in autistic people, particularly those with intellectual disability, is concerning because it means that individuals may not receive timely treatment, leading to debility, poor health-related quality of life, and premature mortality.^23^^,^^50^ Missed diagnoses may be particularly likely when symptoms are subjective (e.g., pain, a feeling of being unwell) rather than observable, making them harder to describe to a service provider. Interoception (the sense that allows us to recognise our internal body states) may not work typically in autistic people.^81^ Atypical interoception may mean that bodily sensations (e.g., thirst, hunger, temperature changes, or the onset of illness) are less likely to trigger subjective awareness meaning that symptoms go unnoticed. Diagnostic overshadowing of physical health by mental health conditions may also contribute to missed diagnoses.^17^

People with moderate to severe intellectual disability may be unable to label or describe pain or discomfort, or know to tell someone about it.^82^ Pain may trigger behaviours that challenge or self-injury, e.g., “headbanging to the point of open wounds or bruising that is caused by pain from an unidentified but treatable health condition such as gastro-oesophageal reflux”.^82^ This can lead to diagnostic overshadowing, where pain-related behaviours are assumed to be part of the person's autism or intellectual disability; or assumed to reflect a mental health problem.^82^ Tools such as the BeWell checklist have been developed to raise awareness of pain as a potential factor underpinning changes in behaviour in people with intellectual disability.^83^ More work is needed to develop and validate assessment tools to identify pain suitable for people of all ages who communicate differently, particularly autistic people with intellectual disability. Future research should also consider how the presence of co-occurring conditions such as cerebral palsy and rare genetic conditions impact diagnoses of common health conditions.

### Strengths and limitations

This study has several strengths and limitations. The autistic and matched comparison participants were registered at the same general practices, meaning that between-practice variation in recording of health conditions would have affected both groups equally. The key limitation of the study is that no large-scale epidemiological studies have been conducted to establish the true rates of the studied conditions in diagnosed autistic people with and without intellectual disability using an active case-finding approach. In the present study, we did not have data on symptom severity for the health conditions studied. Further studies are needed using this approach to provide more conclusive evidence for underdiagnosis. Based on the present findings, we cannot tease apart the extent to which the observed differences are due to differences in the true prevalence of the studied conditions vs. differences in healthcare access or detection of health problems.

In this study, we have made inferences about the true rates of the studied health conditions partly from surveys and volunteer studies where the presence/absence of health conditions was actively identified. However, these studies suffer from their own methodological limitations. Recruitment and participation biases might lead to individuals with more significant difficulties, or particular types of unmet health needs, to preferentially enrol. Whilst medical records likely underestimate true prevalence, it is possible that surveys may over-report rates due to sampling biases. Therefore, it is possible that difference in rates between the medical record and other data sources may be over-stated in this study where these inferences are based on data from volunteer studies.

A further limitation of this study is generalisability. Only a small proportion of autistic adults living in the UK have been diagnosed.^45^ We estimate that only around one in 10 people who would meet current criteria for autism had been diagnosed across the time-period studied in this cohort. Undiagnosed autistic people may have different support needs compared diagnosed autistic people, impacting their health needs. Autistic people living independently in the community are more likely to not have had their autism diagnosed.^84^^,^^85^ These individuals may have fewer communication challenges; but may have additional support needs in other domains, or co-occurring conditions that overshadow their autism. The high level of underdiagnosis also meant that, due to sample size, there was less power to detect more subtle differences, particularly for autistic people (women in particular) with intellectual disability.

It is also likely that many people with milder forms of intellectual disability have not had their intellectual disability diagnosed and recorded in their medical records.^86^ This could bias the estimates for autistic people with intellectual disability towards those with more severe forms of intellectual disability, and could also mean that some participants with undiagnosed mild intellectual disability were included in the autism without intellectual disability group. Therefore, we cannot assume that these findings generalise to all autistic people with and without intellectual disability (both diagnosed and undiagnosed). Community-based studies using active identification approaches are needed to capture both diagnosed and undiagnosed individuals.

In the present study, we did not adjust for socioeconomic status (SES), as we believe that SES is on the causal pathway of poorer healthcare access in autistic people, which would affect incidence rates. By virtue of being more likely to experience unemployment and under-employment, autistic people are more likely to experience socioeconomic deprivation, contributing to health inequalities.^87^ We were also unable to explore rates of diagnoses of health conditions experienced by gender diverse autistic people, as gender diversity was not coded in the database.

Race/ethnicity is a potential confounder. This was not included in our models due to the non-random missingness of ethnicity information captured in primary care data.^88^ Data from England indicate that different ethnic groups differ in their likelihood of accessing autism services and being identified and diagnosed as autistic,^89^^,^^90^ meaning that ethnicity may impact both the likelihood of being diagnosed and of experiencing health inequalities. Research using advanced methods to identify missing ethnicities e.g.,^88^ is urgently needed to address questions around autism, ethnicity and healthcare access using data from primary care.

A further limitation is that the present findings may not generalise to other countries, time periods, or settings where more active screening approaches (e.g., checklists) are used to identify symptoms. New initiatives such as tailored annual health checks for autistic people, which are currently being trialled in the UK,^91^ may lead to increased detection of common health conditions, potentially leading to higher rates of recording in the future. Evidence-based approaches that lead to greater identification of health conditions have the potential to reduce delayed diagnosis and missed treatment opportunities.

### Conclusions and implications

We investigated rates of new diagnoses of common health conditions for diagnosed autistic people and a matched population-based comparison sample. Autistic people with and without intellectual disability had a more than two-fold rate increase in self-harm (including self-injurious behaviour), indicating disproportionately unmet mental health and/or physical or sensory-related support needs. While studies are not directly comparable, for autistic people without intellectual disability, the rate of new anxiety and depression diagnoses relative to the comparison group was not nearly as high as would be expected given the difference in the frequency of these conditions apparent in survey studies, suggesting that common mental health conditions may be under-diagnosed. Comparable or modestly lower rates of physical health diagnoses may also suggest under-diagnosis, given the evidence for higher frequency of the studied physical health conditions in autistic people without intellectual disability.

Autistic people with intellectual disability were less likely than the comparison group to have records of newly-identified depression, and new records of migraine, neck or back pain, or gynaecological conditions, when codes indexing premenstrual dysphoria were excluded. The present results could indicate under-diagnosis of these, and potentially other, painful and debilitating conditions; though studies in which symptoms are actively measured and compared to rates of diagnoses are needed to confirm this. Together with other evidence for health inequalities affecting autistic people, the present findings raise the possibility that pain, discomfort, and distress may be a common experience, particularly for those with intellectual disability; and that potentially serious conditions may go undetected and untreated, contributing to avoidable deaths e.g.^23^^,^^92^ Advocacy efforts by autistic people and their supporters are focusing on promoting greater understanding of autistic healthcare needs, in particular, better identification of underlying health problems, and the importance of offering time and being receptive to autistic people's communication about their subjective states.^93^ Proactive efforts are needed to detect and treat mental and physical health conditions in autistic people with and without intellectual disability to reduce health inequalities, avoidable suffering, and premature mortality.

## Contributors

EO, DL, RC, CC, FH, JM, JBu, MR, RS, JS, & WM conceived of the study. EO and JS accessed and verified the data. EO undertook the analysis. All authors interpreted the findings. EO wrote the first draft of the manuscript; all other authors revised the manuscript for critically important content and approved the final version. The corresponding author attests that all listed authors meet authorship criteria and that no others meeting the criteria have been omitted. All authors accept responsibility to submit for publication.

## Data sharing statement

Individual participant data cannot be shared.

## Ethical approval

IMRD holds ethical approval to collect and supply data for research purposes from the NHS London—South East Research Ethics Committee (reference 18/LO/0441). Use of the IMRD for this study was obtained and approved by IQVIA World Publications Scientific Review Committee in June 2021 (reference 21SRC014).

## Declaration of interests

SH, DL, RC, CC, CEB, FH, JM, JBr, RS, JS, & WM declare no support from any organisation for the submitted work. EO received a post-doctoral fellowship from the Dunhill Medical Trust which funded completion of the work. DM was supported by NIHR as an In-Practice Fellow [NIHR301988]. MR was supported by the Medical Research Council [MC_UU_00019/1] and [MC_UU_00019/3] and JBu was supported by the Royal College of Psychiatrists. JS received funding from the ESRC and NIHR. WM is involved in unrelated projects funded by ESRC, NIHR, MRC, ERC, Sarepta Therapeutics, and Autistica, and received royalties from Jessica Kingsley publishers and a staff training fee from Jazz Pharma. SH was funded by NIHR. FH is part-funded by the NIHR Biomedical Research Centre at South London and Maudsley NHS Foundation Trust and King's College London. RC received funding from NIHR. DM has received payment from EMIS/patient info for writing patient- and professional-facing material for topics unrelated to this manuscript.

All authors declare that they have no financial relationships with any organisations that might have an interest in the submitted work in the previous three years, and no other relationships or activities that could appear to have influenced the submitted work. The views expressed are those of the authors and not necessarily those of the NHS, the NIHR or the Department of Health and Social Care.

## Appendix A Supplementary data

eFigures

eTable

Codelists

## Footnotes

m We use identity-first language (i.e. “autistic person”) based on guidelines published by the National Autistic Society.
