# Supplementary material for: Diagnosis of common health conditions among autistic adults in the UK: evidence from a matched cohort study
Source: Lancet Reg Health Eur. 2024 May 3;41:100907. doi: 10.1016/j.lanepe.2024.100907 (PMC11306212; doi:10.1016/j.lanepe.2024.100907)
Supplement: eFigures [file mmc1.docx]

**Supplementary Figures for “Diagnosis of common health conditions in UK autistic adults: evidence from a matched cohort study”**

Contents

[eFigure 1: Flow diagram for practice/ participant exclusions 2](#_Toc160538400)

[eFigure 2: Simplified schematic description of steps to identify the matched groups & start dates 3](#_Toc160538401)

[eFigure 3: Identification of read codes for autism 5](#_Toc160538402)

[References 6](#_Toc160538403)

## eFigure 1: Flow diagram for practice/ participant exclusions

| 794 practices |  | 2 excluded: Missing Acceptable Computer Usage/ Acceptable Mortality Recording date |
| --- | --- | --- |
|  |  |  |
| 792 practices  18,241,856 people |  | 2,517,068 excluded due to poor record quality or not being permanently registered. |
|  |  |  |
| 15,724,788 people |  | 6,163,338 excluded due to no data after the point that the practice met data quality thresholds for electronic recording of patient data, no person-time after 01/01/2000, and/or no person-time after 18^th^ birthday. |
|  |  |  |
| 9,561,450 people |  | 34,821 excluded due to having diagnosed ID prior to cohort entry and no subsequent autism diagnosis (n = 34,648), or a record of autism with no date (n = 173) |
|  |  |  |
| 22,303 people with an autism diagnosis prior to, or during follow-up |  | 9,504,311 people with no autism record at any time; 15 people with a record of autism after the end of follow-up. |
|  |  |  |
| **Autististic people with and without intellectual disability**  17,249 people contribute person-time post an autism diagnosis (of whom 1,432 got a subsequent ID diagnosis prior to the end of follow-up. These individuals were excluded from the autism/ID- group, leaving 15,817).  6,486 people contribute person-time post an autism and an ID diagnosis. |  | **Sampling pool for identification of matched participants**  9,504,311 people with no autism record at any time.  3,913 people contribute person-time prior to an autism or an ID diagnosis. |
|  |  |  |
| 22,303 to be matched  191 had insufficient matches |  |  |
|  |  |  |
| **22,112 matched 10:1** |  | **221,120 matches** |

## eFigure 2: Simplified schematic description of steps to identify the matched groups & start dates

The schematics below depict how the matched groups were identified and cohort entry dates assigned . Rows represent different individuals within a primary care practice, registered and contributing data from 2001 up until 2011 or the point at which they left the practice (a hypothetical example to illustrate the process).

**Step 1:** We allocated person-time (after patient registration + 6 months and once data quality thresholds have been met) to one of 4 categories, designated by the different colours:

|  | **2001** | **2002** | **2003** | **2004** | **2005** | **2006** | **2007** | **2008** | **2009** | **2010** |  |  | **Key** |  |  |
| --- | --- | --- | --- | --- | --- | --- | --- | --- | --- | --- | --- | --- | --- | --- | --- |
| **A** | **.** |  |  |  |  | **X** |  |  |  |  |  | **.** | **Date of registration** | | |
| **B** | **.** | **X** |  |  |  | **!** |  |  |  |  |  | **X** | **Autism diagnosis** | | |
| **C** | **.** |  |  |  |  |  |  | **X** |  |  |  | **!** | **Intellectual disability diagnosis** | | |
| **D** | **.** |  |  |  |  |  |  |  |  |  |  |  | **Autism no ID** | | |
| **E** | **.** |  |  | **!** |  |  |  | **X** |  |  |  |  | **Autism with ID** | | |
| **F** | **.** |  |  |  |  |  |  |  |  |  |  |  | ***Potentially* eligible person-time: matched comparison group** | | |
| **G** | **.** |  |  |  |  |  |  | **X!** |  |  |  |  | ***Ineligible person-time*** | | |
| **H** | **.** |  |  |  |  |  |  |  |  |  |  |  |  | | |

*Note: ID = Intellectual disability. B was diagnosed with ID after being diagnosed autistic, so could contribute person-time to both the autism/ID- and to the autism/ID+ groups. However, we decided to exclude the person-time prior to B’s ID diagnosis to avoid biasing the estimates for the autism/ID- group.*

**Step 2:** For each autistic person who had follow-up time without an ID record (A and C), we identified a set of randomly-sampled matched people who were in the database & had neither an autism nor an ID record on the date of the autistic person’s diagnosis. We gave them the same start (“index”) date as their autistic counterpart (the date of the autistic person’s autism diagnosis). Those who went on to get an autism or an ID diagnosis were censored from the comparison group prior to their diagnosis (e.g. participant C).

|  | **2001** | **2002** | **2003** | **2004** | **2005** | **2006** | **2007** | **2008** | **2009** | **2010** |  | |  |  |  |  |
| --- | --- | --- | --- | --- | --- | --- | --- | --- | --- | --- | --- | --- | --- | --- | --- | --- |
| **A** | **.** |  |  |  |  | **X** |  |  |  |  |  | |  |  |  |  |
| **C** | **.** |  |  |  |  |  |  |  |  |  |  | |  |  |  |  |
| **Z** | **.** |  |  |  |  |  |  |  |  |  |  | |  |  |  |  |
| **C** | **.** |  |  |  |  |  |  | **X** |  |  |  |  |  |  |  |  |
| **X** | **.** |  |  |  |  |  |  |  |  |  |  |  |  |  |  |  |
| **P** | **.** |  |  |  |  |  |  |  |  |  |  | |  |  |  |  |

**Step 3:** For each autistic person who had follow-up time *with* a concurrent ID record (B, E, and G), we sampled a new set of matches by identifying people who were in the database & had neither an autism nor an ID record on the date of the autistic person’s diagnosis. We gave them the same start (“index”) date as their autistic counterpart (the latest of the date of their autism or ID diagnosis).

|  | **2001** | **2002** | **2003** | **2004** | **2005** | **2006** | **2007** | **2008** | **2009** | **2010** |  |
| --- | --- | --- | --- | --- | --- | --- | --- | --- | --- | --- | --- |
| **B** | **.** | **X** |  |  |  | **!** |  |  |  |  |  |
| **U** | **.** |  |  |  |  |  |  |  |  |  |  |
| **M** | **.** |  |  |  |  |  |  |  |  |  |  |
| **E** | **.** |  |  | **!** |  |  |  | **X** |  |  |  |
| **S** | **.** |  |  |  |  |  |  |  |  |  |  |
| **X** | **.** |  |  |  |  |  |  |  |  |  |  |
| **G** | **.** |  |  |  |  |  |  | **X!** |  |  |  |
| **L** | **.** |  |  |  |  |  |  |  |  |  |  |
| **K** | **.** |  |  |  |  |  |  |  |  |  |  |

## eFigure 3: Identification of read codes for autism

12 search terms based on Snowmed codes

**21 Read codes identified**

Alfageh et al., 2020

Sheehan et al., 2015

**30 Read codes**

**29 Read codes** present in **more than one list**.

**2 out of the 4 Read codes** that were present in **one list only** were a different spelling of a code present in **more than one list**.

Code for Rett syndrome was excluded given its exclusion from autism spectrum disorder in ICD-11

**30 Read codes retained in the final list**

**29 Read codes**

27 Snowmed codes

Note: The number of Read codes indicates the number of codes drawn from the source list for which there were one or more individuals in the IMRD database with a record of that Read code. Snowmed codes refer to a revised clinical coding system that is in the process of replacing Read codes. In total, 30 codes were considered indicative of an autism diagnosis in the present study. The final list was checked and approved by clinical members of the team. The list included codes mapping to ICD-10 and ICD-11 categories indicating an autism spectrum condition, plus historically-used terms, such as “Kanner’s syndrome”, to capture individuals diagnosed autistic before recent diagnostic manuals replaced these terms with more inclusive categories.

## References

Alfageh BH, Man KKC, Besag FMC, Alhawassi TM, Wong ICK, Brauer R. Psychotropic Medication Prescribing for Neuropsychiatric Comorbidities in Individuals Diagnosed with Autism Spectrum Disorder (ASD) in the UK. J Autism Dev Disord 2020; 50: 625–33.

Sheehan R, Hassiotis A, Walters K, Osborn D, Strydom A, Horsfall L. Mental illness, challenging behaviour, and psychotropic drug prescribing in people with intellectual disability: UK population based cohort study. BMJ 2015; : h4326.
