# Supplementary material for: Diagnosis of common health conditions among autistic adults in the UK: evidence from a matched cohort study
Source: Lancet Reg Health Eur. 2024 May 3;41:100907. doi: 10.1016/j.lanepe.2024.100907 (PMC11306212; doi:10.1016/j.lanepe.2024.100907)
Supplement: eTable [file mmc2.docx]

**eTables: Diagnosis of common health conditions in UK autistic adults: evidence from a matched cohort study**

Contents

[eTable 1: Crude incidence of common health conditions in autistic people with and without intellectual disability (ID) and their respective comparison groups: sensitivity analysis 2](#_Toc162351592)

## eTable 1: Crude incidence of common health conditions in autistic people with and without intellectual disability (ID) and their respective comparison groups: sensitivity analysis

| New records of a given condition during follow-up | **Autistic people without ID** | | **Matched comparison group** | |
| --- | --- | --- | --- | --- |
|  | N with no prior record who were diagnosed during follow-up | Crude incidence per 10,000 person-years (95% CI) | N with no prior record who were diagnosed during follow-up | Crude incidence per 10,000 person-years (95% CI) |
| Gynaecological disorders excluding premenstrual tension* | 64/3423 | 68.42 (52.69 - 87.36) | 714/34461 | 69.16 (64.18 - 74.42) |
| Self-harm excluding self-injurious behaviour | 204/14182 | 45.46 (39.43 - 52.14) | 1142/152785 | 21.81 (20.56 - 23.11) |
| Self-harm excluding potentially unintentional acts (e.g. overdoses, unless intent specified). | 124/14790 | 26.50 (22.04 - 31.59) | 503/154910 | 9.45 (8.65 - 10.32) |
|  | **Autistic people with ID** | | **Matched comparison group** | |
|  | N with no prior record who were diagnosed during follow-up | Crude incidence per 10,000 person-years (95% CI) | N with no prior record who were diagnosed during follow-up | Crude incidence per 10,000 person-years (95% CI) |
| Gynaecological disorders excluding premenstrual tension* | 23/1540 | 34.95 (22.16 - 52.44) | 398/14935 | 63.45 (57.37 - 70.00) |
| Self-harm excluding self-injurious behaviour | 96/6074 | 36.90 (29.89 - 45.06) | 530/62429 | 19.54 (17.91 - 21.28) |
| Self-harm excluding potentially unintentional acts (e.g. overdoses, unless intent specified). | 78/6162 | 29.74 (23.51 - 37.12) | 238/63447 | 8.62 (7.56 - 9.78) |

Note: *females only.
